# Generalized Pose Decoupled Network for Unsupervised 3D Skeleton Sequence-Based Action Representation Learning

**DOI:** 10.34133/cbsystems.0002

**Published:** 2022-12-30

**Authors:** Mengyuan Liu, Fanyang Meng, Yongsheng Liang

**Affiliations:** ^1^ Key Laboratory of Machine Perception, Peking University, Shenzhen Graduate School, Shenzhen, China.; ^2^ Peng Cheng Laboratory, Shenzhen, China.; ^3^ Harbin Institute of Technology, Harbin, China.

## Abstract

Human action representation is derived from the description of human shape and motion. The traditional unsupervised 3-dimensional (3D) human action representation learning method uses a recurrent neural network (RNN)-based autoencoder to reconstruct the input pose sequence and then takes the midlevel feature of the autoencoder as representation. Although RNN can implicitly learn a certain amount of motion information, the extracted representation mainly describes the human shape and is insufficient to describe motion information. Therefore, we first present a handcrafted motion feature called pose flow to guide the reconstruction of the autoencoder, whose midlevel feature is expected to describe motion information. The performance is limited as we observe that actions can be distinctive in either motion direction or motion norm. For example, we can distinguish “sitting down” and “standing up” from motion direction yet distinguish “running” and “jogging” from motion norm. In these cases, it is difficult to learn distinctive features from pose flow where direction and norm are mixed. To this end, we present an explicit pose decoupled flow network (PDF-E) to learn from direction and norm in a multi-task learning framework, where 1 encoder is used to generate representation and 2 decoders are used to generating direction and norm, respectively. Further, we use reconstructing the input pose sequence as an additional constraint and present a generalized PDF network (PDF-G) to learn both motion and shape information, which achieves state-of-the-art performances on large-scale and challenging 3D action recognition datasets including the NTU RGB+D 60 dataset and NTU RGB+D 120 dataset.

## Introduction

Human action recognition is a core and fundamental task in the field of computer vision [1], which has wide potential applications in intelligent surveillance, health care, and human–robot interaction [2–4]. For example, a robot needs to understand the command conveyed through human actions before it can naturally interact with human beings. Traditionally, human action recognition methods rely on RGB videos [5,6], but the performance is often not satisfactory because of the lack of depth cues. With the widespread of depth sensors, e.g., Microsoft Kinect [7], recent methods focus on 3-dimensional (3D) human action recognition with depth data, including raw depth sequence [8] and 3D pose sequence [9]. Both types of depth data are provided by depth sensor, where the 3D pose sequence is estimated from depth sequence with a robust pose estimation method [10]. Compared with the depth sequence, the pose sequence just contains compact and meaningful representation via the body, which facilities application in lightweight yet accurate human action recognition systems.

Under the supervision of substantial human-labeled pose sequences, 3D action recognition using pose sequences has achieved high success in distinguishing similar actions with learned representations [11,12]. However, collecting labeled data is usually time-consuming and needs huge manual labor. As an alternative, an unsupervised 3D action recognition task was presented to recognize actions with representations learned without any action annotations. Following the formulation of self-supervised learning [13], a new and useful type of unsupervised learning, the key to the problem is to design a proper supervision signal that can drive the network to learn distinctive representation. Previous methods [14–16] use the original pose sequence as a supervision signal and use a basic autoencoder framework [17] to reconstruct the original pose sequence. The final midlevel latent code generated by the encoder is used as the representation, in the sense that the compact code needs to represent the original pose sequence to reconstruct the whole signal. However, these methods ignore motion information that provides a strong cue for inferring action type. Taking 2 similar actions “clapping” and “rubbing two hands” as an example, these 2 actions share a similar whole-body state except for the slight differences in human hand movements. Representations learned by reconstructing the original pose sequence inevitably ignore the slight differences that are treated equally with reconstruction noise contained in pose sequences, leading to confusion about similar actions.

**Fig. 1.**
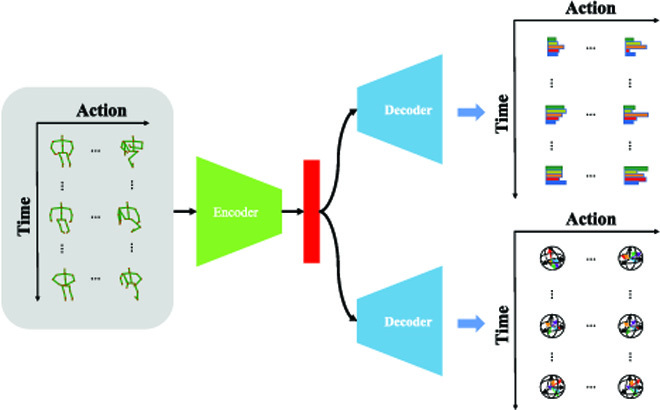
General idea of our pose decoupled flow (PDF) network. Different from the basic autoencoder framework that aims to reconstruct its original input, we leverage motion cues from pose sequences and generate the direction and norm of pose flow as supervision signal, which guides our PDF network to learn distinctive action representation.

To solve this problem, we introduce the concept of pose flow, namely, the optical flow of the pose sequence, as the reconstruction target of the basic autoencoder framework, which intends to drive the latent code to learn more distinguishable motion features. Instead of using the traditional interframe optical flow, the pose flow is defined as the movements between each pose and a reference pose. Usually, the average pooling result of the pose sequence along the temporal axis can be used as the reference pose. The movements between each pose and the reference pose are expected to be more obvious than interframe movements, indicating a more stable signal. We further boost the performance of pose flow by decoupling it as pose magnitude flow and pose orientation flow, which simply denotes the magnitude and orientation of movements in pose sequences. Both pose magnitude flow and pose orientation flow are abbreviated as pose decoupled flow (PDF). In contrast to PDF, disentangled factors including magnitude and orientation information are coupled in pose flow, which prevents the autoencoder framework from learning a more distinctive representation. Therefore, we take advantage of PDF to supervise our proposed PDF network, which uses the same encoder with a basic autoencoder framework and uses 2 split decoders to reconstruct PDF as a multitask learning problem, shown in Fig. 1. In addition, using the original pose sequence to implement additional constraints between decoders and adopting an adaptive training strategy for a multitask learning framework, we further boost the representation learned from PDF, which achieves state-of-the-art performances on 2 benchmark datasets. In general, our main contributions are as follows.

• Compared with previous methods, we present a generalized PDF network (PDF-G) to learn 3D action representation, which contains distinctive motion information. Instead of reconstructing pose flow, learning from decoupled factors enables PDF-G to ignore either direction or norm that barely contains distinctive features in certain action types.
• We use shape information to serve as regularization for PDF-G, which enables PDF-G to learn from both motion and shape features. PDF-G achieves notable improvements over state-of-the-art unsupervised and some supervised methods.

Supervised and unsupervised 3D action recognition using pose sequences are most related to our work. In the following, we review supervised methods and introduce unsupervised methods in detail. Furthermore, the main differences between unsupervised methods and our method are discussed.

According to the type of features, supervised 3D action recognition methods can be divided into handcrafted feature-based [18,19] and deep feature-based methods, where deep features can be further extracted by recurrent neural network (RNN) [20–22], convolutional neural network (CNN) [23–25], and graph convolutional network (GCN) [11,26,27]. Compared with handcrafted features, deep features are learned by neural networks, e.g., RNN, CNN, and GCN, to distinguish labeled pose sequences with similar appearances. Especially using GCN, deep features [11,26,27] outperform handcrafted features [18,19] by a large margin.

Compared with supervised 3D action recognition, unsupervised learning action representation for 3D action recognition is challenging, and few attempts have been tried. Zheng et al. [14] use an autoencoder framework to regenerate the input pose sequence, and the final state of the encoder hidden representation is applied for action recognition. Different from the basic autoencoder framework that uses mean square error (MSE) loss, a discriminator is adopted to discriminate whether the regeneration is accurate. Su et al. [16] reach enhanced performance compared to Zheng et al. [14] due to novel training strategies that weaken the decoder and that strengthen the training of the encoder. Instead of using a basic autoencoder framework, Nie et al. [15] present a Siamese denoising autoencoder framework to learn action representation by disentangling the pose-dependent and view-dependent feature from the human skeleton data, and the 2 disentangled features are concatenated as the representation of the 3D pose sequence. Beyond reconstructing pose sequences, Lin et al. [28] recently integrate motion prediction, jigsaw puzzle recognition, and contrastive learning to learn action representation from different aspects.

Different from previous unsupervised learning methods that usually reconstruct the original pose sequence [14–16] or predict partial original pose sequence [28], this paper focuses on using pose flow and PDF as motion cues to supervise autoencoder framework to learn more distinctive 3D action representation.

## Methodology

Our target is to learn 3D action representation from unlabeled pose sequences. The basic autoencoder framework reconstructs the original pose sequence. To ensure a fair comparison with our network, we implement a basic autoencoder framework with 2 decoders. Actually, the performance of 2 decoders and 1 decoder show no obvious differences. Our explicit PDF (PDF-E) network directly reconstructs PDF, including pose magnitude flow and pose orientation flow. Our implicit PDF (PDF-I) network reconstructs midlevel variables, upon which we can reconstruct PDF. Both variables ares regularized with additional constraint loss. Compared with the implicit version, our PDF-G network uses the original pose sequence to implement the constraint loss, thus providing stronger regulation to the PDF network.

For a given pose sequence, its corresponding PDF is generated by the supervision signal generation part, which is used as the ground truth of our PDF network. Inspired by the traditional autoencoder framework, our PDF network uses an encoder to compress a given pose sequence as a compact latent code and then uses 2 separate decoders to estimate 2 components of PDF, namely, pose magnitude flow and pose orientation flow. To optimize the network, we use MSE loss to enforce the similarity between the estimated pose magnitude flow and its corresponding ground truth and use cosine distance as a loss to enforce the similarity between the direction of the estimated pose orientation flow and its corresponding ground truth. Moreover, we use constraint loss to regularize the PDF network and use an adaptive weighting strategy to balance the optimization process of the 2 decoders. In the testing step, only the trained encoder is used to extract the latent code from any input pose sequence, and the latent code is used as the final representation, which can be used for recognizing 3D actions with many classifiers such as the simple *k*-nearest neighbor (KNN) method. In the following, we introduce our PDF network with 3 sections including supervision signal generation, PDF network, and network optimization.

### Supervision signal generation

As an increasing and effective branch of unsupervised learning, self-supervised learning converts the unsupervised learning task into a supervised learning task, which has already achieved wider success. The core idea is to generate a supervision signal from unlabeled data and then use the unlabeled data as input and use the generated supervision signal as a “label” to train a network. We use self-supervised learning to train a network that is able to extract action representation, where different supervision signals dramatically affect the training process. It remains an open problem to choose proper supervision signals that drive the network to learn distinctive action features. In the following, different supervision signals are discussed in detail.

#### Pose sequence

Given a pose sequence $P={\left\{P_{t}\right\}}_{t=1}^{T}$, which contains *T* poses organized according to natural temporal order. The *t*th pose *P_t_* is denoted as ${\left\{J_{t}^{n}\right\}}_{n=1}^{N}$, which contains *N* joints. The *n*th joint $J_{t}^{n}$ is denoted as $\left\{x_{t}^{n},y_{t}^{n},z_{t}^{n}\right\}\in {\mathbb{R}}^{3}$, which is a coordinate point in 3D space. Previous methods use pose sequence *P* as a supervision signal. The network, which acts like an identity function, takes *P* as input and is expected to reconstruct the input itself.

#### Consecutive pose flow

The above assumption is that good representation contains whole information to reconstruct the original signal. However, using pose sequence *P* as a supervision signal has 2 main shortcomings. First, the pose sequence will bring the noise to the final learned representation, as the representation is optimized to reconstruct the original pose sequence including its contained noise. Because the pose sequence is usually estimated from the depth sequence captured by the depth sensor, both the depth sensor and estimation method bring the noise to the estimated pose sequence. Second, the learned representation only preserves the main components of the original pose sequence, while these components are not distinctive to distinguish similar actions.

Therefore, we use motion information instead of pose sequence as a supervision signal. Different from the original pose sequence, which can be treated as static information, motion information extracted from the pose sequence benefits the recognition of visual similar actions. Here, we refer to the motion information as pose flow, short for pose-based optical flow. This concept is inspired by optical flow, which is widely used in the traditional video-based human action analysis field. Similar to optical flow, which is calculated by the subtraction of consecutive RGB frames, we define pose flow *F* as Δ*P*, where Δ calculates the subtraction between temporal consecutive poses. The definition of pose flow here is called consecutive pose flow (CPF).

#### Reference pose flow

Robust optical flow can be extracted from consecutive RGB frames because whole pixel values except for others on motion regions are quite stable, while CPF can be extremely noisy, as joints in both motion and nonmotion regions are not stable. Given 2 noisy poses, the optical flow between them suffers noises from both.

To alleviate the above problem, we present the concept of reference pose as $1/T*\sum_{t=1}^{T}P_{t}$, which means that the average of whole poses is expected to be more stable than each pose. By comparing each pose with the reference pose, we define pose flow as:

$$F=P-\frac{1}{T}\sum_{t=1}^{T}P_{t}$$ (1)

which represents movements between each pose and the reference pose. Here, the definition of pose flow is called the reference pose flow (RPF). The RPF contains less noise than the CPF for 2 reasons. First, CPF is calculated with 2 noisy signals, namely, 2 poses, while RPF is calculated with 1 noisy signal and 1 stable signal, namely, 1 pose and 1 reference pose. Second, the motion magnitude between consecutive poses is usually relatively smaller than that between 1 pose and 1 reference pose.

#### Pose decoupled flow

When we directly use either CPF or RPF as a supervision signal, the network has to estimate both the magnitude and orientation of pose flow at the same time. To decrease the optimization difficulty, we decouple the pose flow estimation task into 2 subtasks, namely, pose magnitude flow estimation and pose orientation flow estimation, where each subtask is handled by 1 dedicated subnetwork.

In the following, we use RPF as an example. Given a pose sequence *P*, we follow Eq. 1 to calculate $F={\left\{{\left\{F_{t}^{n}\right\}}_{t=1}^{T}\right\}}_{n=1}^{N}$, which can be decoupled as:

F=M⋅O (2)

where *M* denotes the pose magnitude flow, *O* denotes the pose orientation flow, and ⋅ means the dot product of 2 vectors. The pose magnitude flow *M* is denoted as:

$$M={\left\{{\left\{\alpha_{t}^{n}\right\}}_{t=1}^{T}\right\}}_{n=1}^{N}\;$$ (3)

which contains scalars indicating the magnitude. The pose orientation flow *O* is denoted as:

$$O={\left\{{\left\{\left[i_{t}^{n},j_{t}^{n},k_{t}^{n}\right]\right\}}_{t=1}^{T}\right\}}_{n=1}^{N}$$ (4)

which are composed of unit vectors indicating the orientation of pose flow. Note that both pose magnitude flow and pose orientation flow are called components of PDF.

### Pose decoupled flow network

Previous methods use pose sequence *P* as supervision signal to train autoencoder framework, which has 1 encoder network ε and 1 decoder network *D*. Specifically, the encoder network takes *P* as input and generates a compact latent code *Z* formulated as:

$$\;Z=\varepsilon \left\{P\right\}$$ (5)

where $Z\in {\mathbb{R}}^{C}$ and is used as the final action representation. Here, the symbol *C* is a fixed scale indicating the length of representation. Then, the decoder network takes *Z* as the input and reconstructs *P* as $\hat{P}$, which is denoted as:

$$\hat{P}=D\left\{Z\right\}$$ (6)

where $\hat{P}$ shares the exact same shape with *P* as the supervision signal; we can simply take *Z* generated by Eq. 5 as input and generate $\hat{F}$ by:

$$\hat{F}=D\left\{Z\right\}$$ (7)

where $\hat{F}$ is the reconstructed pose flow, and the decoder network *D* aims to make $\hat{F}$ and *F* more similar. To decrease of difficulty of directly reconstructing pose flow, we use 2 subnetworks to reconstruct 2 components of pose flow, namely, pose magnitude flow and pose orientation flow.

#### Explicit network

In order to reconstruct components of PDF, we extend the basic autoencoder framework structure and use 2 separate decoders, namely, *D_M_* and *D_O_*, following 1 shared encoder *ε*. Taking *Z* from Eq. 5 as input, we use *D_M_* and *D_O_* to reconstruct pose magnitude flow and pose orientation flow as:

$$\hat{M}=D_{M}\left\{Z\right\}$$ (8)

$$\hat{O}=D_{O}\left\{Z\right\}$$ (9)

where $\hat{M}$ and $\hat{O}$ build the reconstructed pose flow ${\hat{F}}_{e}$ as:

$${\hat{F}}_{e}=\hat{M}\cdot \hat{O}$$ (10)

**Fig. 2.**
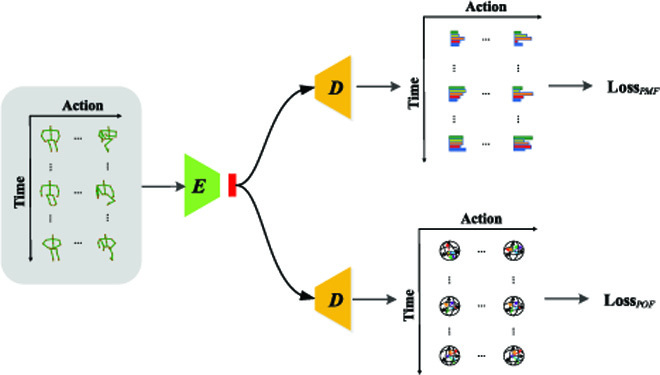
Illustration of our proposed explicit PDF network, where "*E*" denotes encoder and "*D*" denotes decoder.

This type of PDF network, namely, 2 decoders, is called an explicit network (in Fig. 2), because both decoders directly output the targets we need.

#### Implicit network

**Fig. 3.**
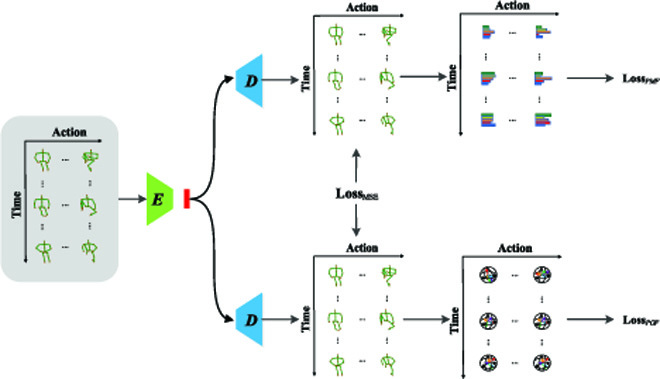
Illustration of our proposed implicit PDF network.

Despite the simplicity of the explicit network, we present an alternative PDF-I network (in Fig. 3), which first use 2 decoders to generate variables, from where pose magnitude flow and pose orientation flow are then inferred. Different from the explicit network where 2 decoders are independent of each other, we constrain the generated midlevel variables to provide regularization to decoders of the implicit network. Specifically, subnetworks *D_M_* and *D_O_* are used to generate variables ${\hat{P}}_{M}$ and ${\hat{P}}_{O}$:

$${\hat{P}}_{M}=D_{M}\left\{Z\right\}$$ (11)

$${\hat{P}}_{O}=D_{O}\left\{Z\right\}$$ (12)

where both variables share the same shape with the original pose sequence *P*, whose physical meaning can be simply interpreted as newly generated pose sequences. To infer pose magnitude flow from ${\hat{P}}_{M}$, we follow Eq. 1 to extract reference flow, which can be decoupled by Eq. 2 to generate ${\hat{M}}_{M}$. To infer pose orientation flow from ${\hat{P}}_{O}$, we follow Eq. 1 to extract reference flow, which can be decoupled by Eq. 2 to generate ${\hat{O}}_{O}$. In general, we can implicitly reconstruct pose flow ${\hat{F}}_{i}$ as:

$${\hat{F}}_{i}={\hat{M}}_{M}\cdot {\hat{O}}_{O}$$ (13)

This type of pose decoupled network, namely, 2 decoders, is called an implicit network because we have to infer from the midlevel variables generated by the network to obtain the targets.

#### Generalized network

**Fig. 4.**
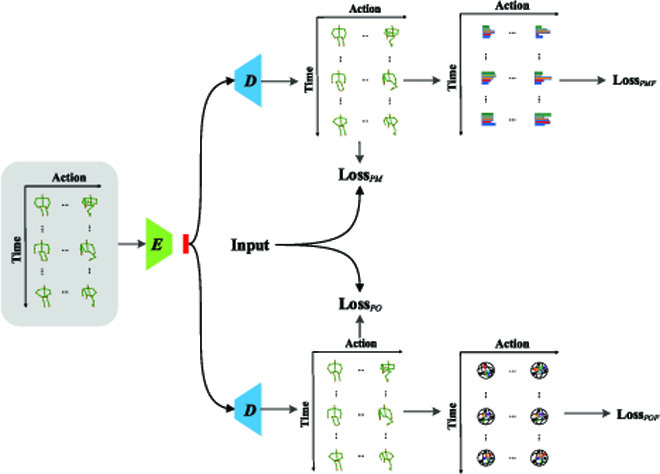
Illustration of our proposed generalized PDF network.

We observe that pose flow reconstructed by our PDF network contains features that are distinctive to recognize similar actions. One possible shortcoming is that pose flow can barely capture subtle motions, especially from noisy pose sequences. For an extreme example, a still pose without any movements is also called an action, which contains no pose flow information. Therefore, we generalize our PDF network to take both pose flow and original pose sequence as reconstruction targets, which achieves state-of-the-art performances. Compared with previous methods that use only pose sequence as a supervision signal, our generalized network (in Fig. 4) uses pose flow as a supervision signal and uses the original pose sequence as a regulation to enhance the network.

### Network optimization

#### Loss function

To optimize basic autoencoder framework, which is used to reconstruct pose sequence, previous methods use loss *L_p_* defined as:

$$L_{p}=\|P-\hat{P}\|{\;}_{l_{2}}$$ (14)

which calculates MSE between the original pose sequence *P* and the reconstructed pose sequence $\hat{P}$. Symbol *l*_2_ means the L2 norm. To optimize the basic autoencoder that is used to reconstruct pose flow, we follow previous methods and define loss *L_f_* as:

$$L_{f}=\|F-\hat{F}\|{\;}_{l_{2}}$$ (15)

which calculates MSE between the original pose flow *F* and the reconstructed pose flow $\hat{F}$.

To optimize our PDF-E network, we have to calculate similarities between the pair of *M* and $\hat{M}$ and the pair of *O* and $\hat{O}$. The loss function *L_e_* is defined as:

$$L_{e}=\omega_{1}\|M-\hat{M}\|{\;}_{l_{2}}+\omega_{2}\frac{1-O\cdot \hat{O}}{2}$$ (16)

where we calculate the MSE between *M* and $\hat{M}$ and calculate the cosine distance between *O* and $\hat{O}$. Because the scope of $O\cdot \hat{O}$ ranges from −1 to 1, we normalize it as $\left(1-O\cdot \hat{O}\right)/2$, which ranges from 0 to 1. Parameters *ω*_1_ and *ω*_2_ are used to balance the scope of 2 loss items.

To optimize our PDF-I network, we have to calculate similarities between the pair of *M* and ${\hat{M}}_{M}$, the pair of *O* and ${\hat{O}}_{O}$, and the pair of ${\hat{P}}_{M}$ and ${\hat{P}}_{O}$. The loss function *L_i_* is defined as:

$$L_{i}=\omega_{1}\|M-{\hat{M}}_{M}\|{\;}_{l_{2}}+\omega_{2}\frac{1-O\cdot {\hat{O}}_{O}}{2}+\omega_{3}{\left\|{\hat{P}}_{M}-{\hat{P}}_{O}\right\|}_{l_{2}}$$ (17)

which contains 3 loss items. Compared with *L_e_*, the additional loss item ${\left\|{\hat{P}}_{M}-{\hat{P}}_{O}\right\|}_{l_{2}}$ denotes the constraint loss between 2 decoders, which drives the generated variables as close as possible. Symbol *ω*_3_ balances the importance between constraint loss and reconstruction losses. To optimize our PDF-G network, we define the loss function *L_g_* as:

$$L_{g}=\omega_{1}\|M-{\hat{M}}_{M}\|{\;}_{l_{2}}+\omega_{2}\frac{1-O\cdot {\hat{O}}_{O}}{2}+\omega_{3}L_{{\hat{P}}_{M}}+\omega_{4}L_{{\hat{P}}_{O}}$$ (18)

where $L_{{\hat{P}}_{M}}={\left\|{\hat{P}}_{M}-P\right\|}_{l_{2}}$ and $L_{{\hat{P}}_{O}}={\left\|{\hat{P}}_{O}-P\right\|}_{l_{2}}$, which denote constraint loss implemented by using the original pose sequence. Other symbols are similarly defined according to the loss function *L_i_*. Our PDF network belongs to the category of multitask learning framework, where subtasks are weighted by hyperparameters, i.e., *ω*_1_ and *ω*_2_ in our case, which is expensive to tune. We apply an adaptive training strategy as a more convenient approach to learning the dynamic optimal weights during the training step [29]. Specifically, we derive a multitask loss *L_e_* as:

$$L_{e}=\frac{1}{2\delta_{1}^{2}}{\left\|M-\hat{M}\right\|}_{l_{2}}+\frac{1}{2\delta_{2}^{2}}\frac{1-O\cdot \hat{O}}{2}+\log\delta_{1}\delta_{2}$$ (19)

which maximizes the Gaussian likelihood with homoscedastic uncertainty. Losses *L_i_* and *Lg* can be redefined in a similar manner.

### Unsupervised learning for recognition

We evaluate our learned action representation in an unsupervised manner for 3D action recognition. The whole evaluation procedure follows the previous method [16]. Specifically, we first extract action representation, a fixed-length feature vector, with our proposed network from each training or testing pose sequence. Then, the KNN classifier (with *k* = 1) is used to assign each testing pose sequence to the closest training pose sequence, whose label is used as the predicted label of the testing pose sequence. Here, we use cosine similarity as the distance metric to compare the similarity of feature vectors.

## Experiments

### Evaluation datasets

NTU RGB+D 60 (NTU-60) and NTU RGB+D 120 (NTU-120) are currently very large-scale and challenging datasets for evaluating 3D pose-based action recognition tasks. Samples from these datasets contain high variability in various aspects. For example, the same type of action can be performed by different subjects and different environments recorded by cameras from different views, which facilitates the comparison of different representations.

The NTU-60 dataset contains 60 action types performed by 40 subjects, generating 56,880 pose sequences. Following Su et al. [16], we use Cross Subject (CSub) and Cross View (CView) protocols for evaluation. Under the CSub protocol, the training and testing sets have 40,320 and 16,560 pose sequences, respectively. Under the CView protocol, pose sequences recorded using cameras 2 and 3 are used for training, and the remaining pose sequences recorded using camera 1 are used for testing. In this case, the training and testing sets have 37,920 and 18,960 pose sequences, respectively.

The NTU-120 dataset contains 114,480 pose sequences generated by 106 subjects performing 120 action types observed from 155 views. Following Rao et al. [30], we use CSub and Cross Setup (CSet) protocols for evaluation.

### Experimental settings

Both NTU-60 and NTU-120 datasets are captured using Kinect V2 cameras concurrently, generating RGB videos, infrared videos, depth sequences, and 3D pose sequences estimated from depth sequences. Each pose sequence records the 3D coordinates of 25 body joints at each frame. We present a simple prepossessing method to normalize pose sequences to suppress the effect of noise and view changes. First, null frames in pose sequences are padded with previous frames. Second, the spine joint on each frame is moved to the origin. Finally, the bone between the hip and spine is paralleled to the *z* axis, and the bone tween the right shoulder and the left shoulder is paralleled to the *x* axis. We use normalized pose sequences as inputs for the training network. This normalization step alleviates the training difficulty of the network. To ensure equal comparison with the previous method [16], each pose sequence is sampled to have, at most, 50 frames. We use an Adam optimizer with a learning rate starting from 0.001 and 0.1 decay rate at every 80 iterations. The batch size is 128, and all networks are trained on a Tesla P40 card. The maximum train epoch is 100.

The basic autoencoder framework contains 1 encoder and 1 decoder. The encoder is implemented by 1 single-layer long short-term memory (LSTM), whose output is used as action representation. To ensure a fair comparison with Su et al. [16], the dimension of the representation is set to 256. The decoder contains a single-layer LSTM and an fully connected (FC) layer, which is used to generate output with the expected size. Take a pose sequence with a size of 50 × 3 × 25 as an example, where 50 denotes the number of frames, 3 denotes 3D coordinates, and 25 denotes the number of joints. The encoder takes a vector with a size of 50 × 75 (75 = 3 × 25) as the input and generates a vector with a size of 1 × 256 as the output. Then, we repeat the values of this vector and generate a new vector with a size of 50 × 256. This vector is processed by LSTM in the decoder and outputs a vector with a size of 50 × 256. Finally, the FC layer in the decoder converts the vector to a size of 50 × 75. To ensure a fair comparison with the basic autoencoder framework, the encoder of our network is implemented by 1 single-layer LSTM, and the decoder of our network is implemented by 1 LSTM layer and 1 FC layer.

### Ablation study

Table 1 shows the ablation studies of each component of our method. The baseline method uses pose sequence as a supervision signal to train autoencoder framework. Our proposed CPF and RPF methods use CPF and RPF as supervision signal to train the autoencoder framework, respectively. Our proposed PDF-E and PDF-I methods use PDF as a supervision signal to train the PDF-E and PDF-I network, respectively. Beyond PDF-I, our proposed PDF-G method uses PDF-G network instead. Built upon our PDF-G method, our proposed PDF-G* method uses an enhanced encoder implemented by 2 LSTM layers. Note that the PDF is decoupled by RPF in default.

**Table 1.** Ablation studies of each component of our method on NTU-60 and NTU-120 datasets using different protocols. The symbol “*” denotes an enhanced encoder implemented by 2 LSTM layers. Others use 1 LSTM layer as an encoder by default.

Method	Supervision signal	Network	NTU-60 (CSub)	NTU-60 (CView)	NTU-120 (CSub)	NTU-120 (CSet)
Baseline	Pose sequence	Basic	53.6%	77.8%	42.3%	44.6%
CPF	Consecutive pose flow	Basic	48.3%	71.3%	37.1%	39.7%
RPF	Reference pose flow	Basic	54.8%	77.7%	42.4%	44.8%
PDF-E	Pose decoupled flow	Explicit	56.3%	79.0%	43.4%	47.5%
PDF-I	Pose decoupled flow	Implicit	59.3%	81.0%	47.7%	50.7%
PDF-G	Pose decoupled flow	Generalized	59.7%	81.0%	48.2%	50.9%
PDF-G*	Pose decoupled flow	Generalized	60.4%	81.5%	48.5%	51.3%

#### How to define pose flow?

We provide CPF and RPF as an alternative of pose flow. Table 1 shows that RPF outperforms CPF by more than 5% on both NTU-60 and NTU-120 datasets using different protocols, showing that RPF is a stable supervision signal.

#### Why decouple pose flow?

Different from RPF, which uses pose flow as a supervision signal, PDF-E decouples the pose flow and uses PDF as a supervision signal to train PDF-E network. Here, we simply use 2 decoders to implement the PDF network and focus on the effect of the supervision signal. Table 1 shows that PDF-E achieves an accuracy of 56.3% on the NTU-60 dataset under the CSub protocol, which is 1.5% higher than RPF. Under the CView protocol, PDF-E achieves an accuracy of 79.0% on the NTU-60 dataset, which is 1.3% higher than RPF. We improve the PDF network and present an implicit version, which brings additional constraint loss to regularize 2 decoders. Table 1 shows that the PDF-I network benefits the extraction of proper deep features from PDF. Specifically, PDF-I achieves an accuracy of 59.3% on the NTU-60 dataset under the CSub protocol, which is 4.5% higher than RPF. Under the CView protocol, PDF-I achieves an accuracy of 81.0% on the NTU-60 dataset, which is 3.3% higher than RPF. Similar obvious gains can be found in Table 1.

#### Pose sequence or pose flow?

**Fig. 5.**
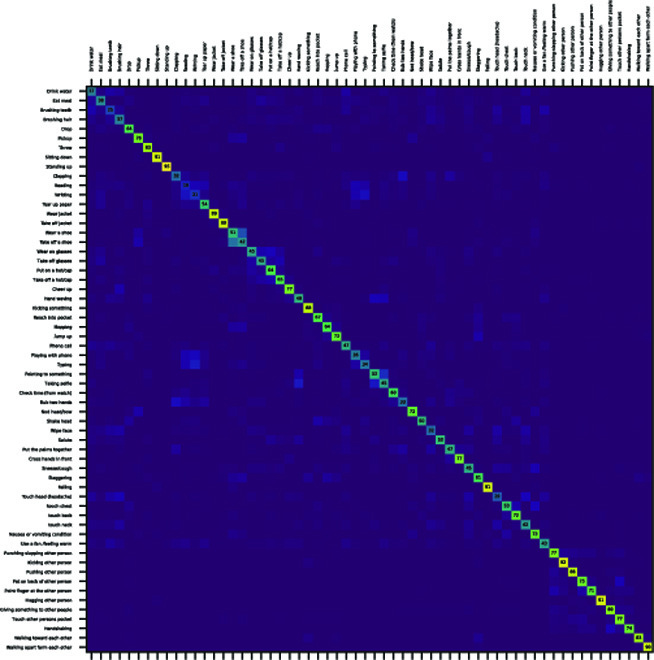
Confusion matrix of our PDF-G method on the NTU-60 dataset using the CSub protocol.

In Table 1, the baseline method uses pose sequence as a supervision signal, and all other methods use pose flow as a supervision signal. The CPF method using noisy CPF obtains worse performances than the baseline. Using RPF instead, RPF achieves slightly higher performances than the baseline. Moreover, PDF-I uses PDF as the supervision signal, which achieves 5.7% higher than the baseline on the NTU-60 dataset under the CSub protocol. Using the generalized version of the PDF network, PDF-G outperforms all previously mentioned methods, e.g., outperforming baseline by 6.1%. We show the confusion matrix of PDF-G in Fig. 5, where similar actions such as “sitting down” and “standing up” can be well distinguished, despite that this pair of actions share extremely similar pose shapes. We list the performance of PDF-G* to show that our method can be combined with more complex encoders to achieve further improvements.

### Comparison with state of the arts

Table 2 shows a comparison of our method with state-of-the-art action recognition methods on the NTU-60 and the NTU-120 datasets. In general, our method outperforms all previous methods under fair evaluation manners. For example, on the NTU-60 dataset using the CSub protocol, our PDF-G method is comparable with the most recent unsupervised pose-based methods, including LongT GAN [14], CAE* [30], P&C FW-AEC [16], and MS2L [28], where our method outperforms them by at least 7%, which is brought by PDF to drive the network to learn distinctive motion information. On the NTU-120 dataset, our PDF-G* method is comparable with the most recent CAE* [30] method that uses 2 LSTM layers as the encoder. Our method outperforms CAE* by 2.1% and 0.2% on the CSet and CSub protocols. It is interesting to find that our method outperforms supervised pose-based methods including P-LSTM [32] and Soft RNN [33] by a large margin.

**Table 2.** Comparison of our method with state-of-the-art action recognition methods using supervised pose, unsupervised RGB+D, and unsupervised pose on the NTU-60 dataset.

Method	NTU-60 (CSub)	NTU-60 (CView)	NTU-120 (CSub)	NTU-120 (CSet)
Supervised pose-based				
HOPC [31]	50.1%	52.8%	-	-
HBRNN [20]	59.1%	64.0%	-	-
P-LSTM [32]	62.9%	70.3%	25.5%	26.3%
Soft RNN [33]	-	-	36.3%	44.9%
ST-LSTM [34]	69.2%	77.7%	55.7%	57.9%
VA-RNN-Aug [35]	79.4%	87.6%	-	-
ST-GCN [26]	81.5%	88.3%	-	-
IndRNN [36]	81.8%	88.0%	-	-
HCN [37]	86.5%	91.1%	-	-
PEM [38]	-	-	64.6%	66.9%
AS-GCN [11]	86.8%	94.2%	-	-
ST-GR [39]	86.9%	92.3%	-	-
DGNN [40]	87.5%	94.3%	-	-
2s-AGCN [27]	88.5%	95.1%	82.9%	84.9%
AGC-LSTM [41]	89.2%	95.0%	-	-
MS-G3D [42]	91.5%	96.2%	86.9%	88.4%
Unsupervised RGBD-based				
Shuffle and learn [43]	46.2%	40.9%	-	-
Luo et al. [44]	61.4%	53.2%	-	-
Li et al. [45]	68.1%	63.9%	-	-
Unsupervised pose-based				
LongT GAN [14]	39.1%	48.1%	-	-
CAE* [30]	-	-	48.3%	49.2%
P&C FW-AEC [16]	50.7%	76.1%	-	-
MS2L [28]	52.6%	-	-	-
PDF-G (ours)	59.7%	81.0%	48.2%	50.9%
PDF-G* (ours)	60.4%	81.5%	48.5%	51.3%

## Conclusion and Future Work

Previous 3D action representation learning methods using autoencoder to reconstruct the original pose sequence can barely extract representation with distinctive motion information. To this end, we explicitly model motion information with a handcrafted pose flow feature to guide the autoencoder to directly learn from motion. The comparable performance with previous methods verifies that pose flow can effectively guide the network to learn distinctive motion information. Furthermore, we infer that the mixture of motion direction and motion norm in pose flow limits the distinctive power of extracted representation. Thus, we present a PDF-E network to learn from decoupled direction and norm of pose flow, which outperforms previous methods by a large margin. Moreover, we use additional shape constraint loss to boost the performance of our network to the state-of-the-art methods, which verifies that our network can simultaneously learn distinctive motion and shape information. Our method can be extended to related research fields including pose-based human action retrieval and 1- or few-shot learning for posed-based human action recognition. Because the performances of our method directly depend on pose estimation methods, improving the robustness of our method to noises in pose sequences will be our future work.
